# Nonpharmacological Management of Gastroesophageal Reflux in Preterm Infants

**DOI:** 10.1155/2013/141967

**Published:** 2013-09-01

**Authors:** Luigi Corvaglia, Silvia Martini, Arianna Aceti, Santo Arcuri, Roberto Rossini, Giacomo Faldella

**Affiliations:** ^1^Neonatology and Neonatal Intensive Care Unit, S. Orsola-Malpighi Hospital, Via Massarenti 11, 40138 Bologna, Italy; ^2^Department of Medical and Surgical Sciences (DIMEC), University of Bologna, Via Massarenti 9, 40138 Bologna, Italy

## Abstract

Gastroesophageal reflux (GOR) is very common among preterm infants, due to several physiological mechanisms. Although GOR should not be usually considered a pathological condition, its therapeutic management still represents a controversial issue among neonatologists; pharmacological overtreatment, often unuseful and potentially harmful, is increasingly widespread. Hence, a stepwise approach, firstly promoting conservative strategies such as body positioning, milk thickening, or changes of feeding modalities, should be considered the most advisable choice in preterm infants with GOR. This review focuses on the conservative management of GOR in the preterm population, aiming to provide a complete overview, based on currently available evidence, on potential benefits and adverse effects of nonpharmacological measures. Nonpharmacological management of GOR might represent a useful tool for neonatologists to reduce the use of antireflux medications, which should be limited to selected cases of symptomatic babies.

## 1. Introduction

Gastroesophageal reflux (GOR) is common in preterm infants, with a 22% estimated incidence in babies born before 34 weeks of gestation [1]. Several factors may contribute to its development: relatively abundant milk intakes, the supine posture which promotes the passage of liquid gastric content into oesophagus, the immature oesophageal motility, and the subsequent poor oesophageal clearance of refluxate [2]. Hence, in the preterm population GOR is due to several physiological mechanisms, and it should not be usually considered as pathological. However, in moderate to severe cases, GOR may lead to complications, such as lung aspiration, esophagitis, feeding problems, and failure to thrive [3], therefore prolonging hospital stay [4]; its linkage with apnoeas [5] or chronic lung disease [6–8] is still on debate.

The therapeutic management of GOR still represents a controversial issue among neonatologists. A stepwise approach, promoting at first nonpharmacological interventions such as body positioning, milk thickening, or modifications in feeding modalities, should be considered the most advisable choice to manage GOR in preterm infants [3, 5, 9]; this would allow avoiding pharmacological treatment, which could be limited to those infants who do not benefit from conservative measures or who experience GOR clinical complications [10].

In recent years, however, the empiric use of antireflux medications, both during hospital stay in Neonatal Intensive Care Unit (NICU) and after discharge [11], has substantially increased. Pharmacological therapies have been shown to increase the risk of serious adverse effects in the neonatal population: the use of gastric acid inhibitors, that is, histamine-2 (H2) blockers and proton pump inhibitors (PPI), has been recently linked to increased rates of necrotizing enterocolitis (NEC) [12, 13] and infections (overall infections, sepsis, pneumonia, and urinary tract infections) [14], whereas cisapride administration was proven to provoke a relevant prolongation of QTc [15, 16].

Specific diagnostic investigations should be performed before treatment, in order to assess GOR features, because the prevalence of acid or nonacid GORs has different clinical and therapeutic implications. Specifically, acid reflux, which occurs predominantly in the late postprandial period [17], is reported to play a relevant role in the development of gastroesophageal reflux disease (GORD) [2], whereas nonacid reflux, which is more frequent during the early postprandial period [17], has been proposed as a potential trigger for GOR-related apnoeas in the preterm population [18]. It should be also considered that many pharmacological therapies act specifically on acid GOR, while different conservative strategies have different effects on acid and nonacid GORs.

Due to its ability to both detect acid, weakly acid, and alkaline GORs [17] and assess the physical nature (gaseous, liquid, or mixed) of reflux episodes, combined multiple intraluminal impedance (MII) and pH monitoring is highly effective in detailing GOR features, being superior to pH-metry and MII alone. It is currently considered the best choice to diagnose GOR [3, 19] and to evaluate the efficacy of antireflux therapy [20]. Additionally, a reflux scoring system based on clinical observation and suited for hospitalized preterm infants has been recently developed for GOR diagnosis and management [3]; this questionnaire, however, cannot replace standard diagnostic investigations and needs further validation.

This review focuses on conservative management of GOR in preterm infants, aiming to provide a complete overview on potential benefits and adverse effects of currently available nonpharmacological measures.

## 2. Body Positioning

Body positioning is widely used as a conservative approach to manage GOR in hospitalized preterm infants [1]. Since 1982, when a decrease of GOR was noted in prone neonates [21], several trials tested the efficacy of different body positions on GOR indexes in both term and preterm infants.

The first study on preterm infants dates back to 1999, when Ewer et al. [22] found a relevant improvement in acid reflux indexes (frequency, reflux index, number of refluxes longer than 5 minutes, and duration of the longest episode) at pH monitoring in both prone and left-side positions. The effectiveness of prone position was later confirmed by Bhat et al. [23], who examined by means of pH monitoring a cohort of healthy preterm infants before discharge. Omari et al. [24] combined oesophageal manometry and MII to investigate the efficacy of left versus right lateral position on GOR features in healthy preterm infants; left-side positioning resulted in a significant decrease of transient lower oesophageal sphincter relaxations (TLOSRs), which are known to be the main triggering mechanisms for GOR episodes, whereas right lateral position was associated with a higher number of TLOSRs and liquid refluxes [25].

While pH monitoring is not effective in detecting nonacid GORs, MII alone cannot distinguish between acid and nonacid refluxes; hence, we assessed the effects of body positioning on both acid and nonacid GORs in preterm symptomatic infants combining MII and pH monitoring (pH-MII) [26]. Lower acid and nonacid GOR indexes were observed in both prone and left lateral positions. Particularly, left-side positioning led to a significant decrease of acid GOR episodes during the earlier postprandial period (up to 1 hour and 30 minutes after meal), while the prone posture was more effective to reduce acidic oesophageal exposure in the late postprandial period.

In conclusion, body positioning can be considered an effective strategy to manage both acid and nonacid GORs in preterm infants; improvements of GOR indexes are observed in prone and left lateral positions, whereas supine and right lateral positioning seem to play a worsening effect. However, due to the established risk of sudden infant death syndrome (SIDS) linked to prone positioning [27], this measure is limited to hospitalized babies and should not be applied in symptomatic infants discharged without cardiorespiratory monitoring.

Placing the babies on a head-up slope is a measure frequently adopted in clinical practice [1]. However, head elevation resulted to be ineffective to reduce acid GOR in both prone and supine positions [28, 29]; furthermore, the car seat positioning was found to elicit acid GOR exacerbations [30]. Data on preterm infants, however, are currently lacking. Hence, on the basis of the available evidence, head rising should not be considered an effective strategy to reduce GOR in term infants up to six months of life.

## 3. Feeding Strategies

Feeds frequency (every two, three, or four hours), as well as different feeding modalities, are thought to influence GOR features.

The relationship between feeds frequency and GOR episodes in both term and preterm infants was investigated at first by Omari et al. [2], who observed a positive correlation between the frequency of feedings and the occurrence of nonacid GOR episodes, with a concomitant decrease in the number of acid GOR, which is known to be determinant for the development of GORD [31]. According to the results of this study, it can be hypothesised that frequent, small-volume feeds probably reduce GOR in mildly symptomatic infants with prolonged oesophageal acid exposure but have no benefit for symptomatic babies with predominant nonacid GOR.

Bolus and continuous tube feedings are the most common enteral feeding techniques in NICUs. Clinical practice suggests that changing the feeding method (i.e., shifting from bolus to continuous feeding or vice versa) may represent an effective conservative approach in neonates with symptomatic GOR [3]. Indeed, the permanence of a tube through lower oesophageal sphincter (LOS) is shown to affect the occurrence of GOR [32], while the sudden gastric distension determined by bolus administration can impair LOS continence, thus favouring GOR [5, 33, 34].

The effect of different feeding strategies on GOR in a preterm cohort has been recently assessed by Jadcherla et al. [35] by means of pH-MII. A significant negative correlation between feeding duration and total GOR events, number of nonacid GORs, and time of oesophageal bolus clearance was observed. Consistently with this finding, lowered feeding flow rates (mL/min) yielded a decrease in the same GOR features. Thus, the reduction of feeding flow rate, which results in a prolonged feeding duration, seems to represent a potentially useful strategy for GOR management, especially in those infants with predominant nonacid GOR. However, further trials are needed to confirm these preliminary data.

## 4. Feed Thickening

Thickened feeds, that is, human milk or formula added with thickening agents or commercial antiregurgitation (AR) formulas, are increasingly being used as nonpharmacological treatments of GOR in symptomatic infants [1].

In 2008, Horvath et al. systematically evaluated data from randomized controlled trials performed in term infants on the efficacy of thickened formulas on GOR features, detected with pH monitoring. Despite a significant decrease of symptoms as regurgitations and vomiting and an increase in daily weight gain, thickening agents were ineffective in reducing acid GOR indexes [36]. An improvement of acid GOR features in infants fed a corn starch thickened formula has been reported in only one trial [37]. As for nonacid GOR, a remarkable reduction of reflux episodes, mean oesophageal reflux height, and frequency of regurgitation was observed by Wenzl et al. in association with the use of a galactomannan-thickened formula [38].

Further investigations in larger controlled trials are needed in order to investigate the safety profile of thickening agents. So far, an increase in coughing was noticed in symptomatic babies fed on rice-thickened formulas [39], and a case of an allergic reaction linked with carob gum thickening has been described [40]. Moreover, despite that infants fed formulas thickened with indigestible carbohydrate showed normal growth and nutritional parameters at a 3-month follow-up study [41], the use of carob bean gum, evaluated in vitro, was reported to affect the intestinal absorption of calcium, iron, and zinc more than thickening with digestible carbohydrates [42].

With regard to the preterm population, only a small number of studies on the effectiveness and the safety of thickening agents are currently available. We have previously investigated the efficacy of fortified human milk thickened with precooked starch in a small cohort of preterm symptomatic infants: no improvement in the rates of both acid and nonacid GORs was documented; additionally, a trend toward an increase of the total number of GORs was observed. To the best of our knowledge, this was the first study testing the effectiveness of thickened human milk in preterm infants; on the basis of these preliminary results, starch-thickening of fortified human milk does not appear to be an advisable strategy to reduce GOR in preterm symptomatic infants [43]. Furthermore, a linkage between milk thickening and NEC development has been recently suggested [44, 45]; hence, milk thickening in the preterm population is not recommended before an adequate achievement of feeding tolerance [3].

Commercial thickened formulas are inadequate for the nutritional needs of preterm infants, due to their lower caloric and protein contents and to the lack of long-chain polyunsaturated fatty acids (LCPUFA), which play a relevant role in the structural development of retinal membranes and central nervous system (CNS). In our previous study, a starch-thickened formula tailored on preterms nutritional needs was specifically designed to investigate its efficacy on GOR features in symptomatic preterm infants [46]. The thickened formula, however, was found to be ineffective on nonacid GOR features, evaluated by means of combined pH-MII. This result might be explained by the properties of the amylopectin component, which increases its viscosity at an acid gastric pH, being thereby effective during the late postprandial period, when nonacid GOR does not prevail [17]. Despite the significantly lowered rate of acid GORs, the thickened formula failed to reduce the mean oesophageal acid exposure, which is known to be the main determinant index for the development of GORD [31]. Moreover, when compared to a standard preterm formula, this thickened formula yielded a longer duration of acid GOR episodes, which was probably due to a slower oesophageal clearance. There are no data on the safety of thickened formulas in preterm infants. Hence, as well as human milk thickening, formula thickening seems to be ineffective to reduce GOR in symptomatic preterm infants. Nevertheless, larger controlled trials might be advisable to confirm these preliminary findings and to assess the safety of thickened formulas in the preterm population.

## 5. Hydrolysed Formulas

Hydrolysed protein formulas (HPFs) are reported to reduce gastrointestinal transit time [47], to increase stool frequency [48], and to improve feeding tolerance, therefore leading to an earlier achievement of full enteral feeding [49]. Several mechanisms through which HPFs could act have been proposed [50]; whereas some evidence suggests that HPFs elicit a higher motilin release than standard preterm formulas (SPFs) [51], a study performed on animals has shown that protein hydrolysis diminishes the activity of milk protein-derived opioid receptor agonists (*β*-casomorphins) [52]. The effect of HPFs on gastric emptying, though, is still controversial; while some authors found an improvement [47, 53], others found no difference between HPFs and SPFs [54].

Extensively HPFs (eHPFs) have been shown to improve GOR features in term infants and children symptomatic for GOR [55]. Sensitized infants with cow's milk allergy (CMA) are known to develop gastric dysrhythmia, which can lead to a severe impairment of gastric motor function and delayed gastric emptying, thereby contributing to GOR exacerbation [56, 57]. For this reason, infants with CMA are more likely to suffer from symptoms as regurgitation and vomiting, which appear to be indistinguishable from physiological GOR. Thus, when own mother's milk is not available, the dietary management of GOR recommended in the recent guidelines by the European Society of Pediatric Gastroenterology, Hepatology, and Nutrition includes a 2–4-week trial of eHPF or amino acid based formula [20].

In a recent trial, we have compared the efficacy of an eHPF versus a SPF on GOR features in preterm infants symptomatic for both GOR and feeding intolerance. A significant decrease in total acid GOR episodes and reflux index was observed in newborns fed the eHPF, whereas no difference between the two formulas was found in terms of GOR height and nonacid GOR features [58]. Acid GORs being more frequent in the late postprandial period, the reduction in acid GOR could be attributed to the enhancement of gastric emptying previously reported in premature infants fed eHPFs [47]. This is the first study aiming to evaluate the efficacy of eHPFs on GOR features in preterm infants. According to our preliminary findings, eHPFs are effective in decreasing acid GOR in preterm infants experiencing symptoms of GOR and feeding intolerance. However, the sample size was small, leading to a low power to evaluate the clinical efficacy of the eHPF on GOR symptoms. Furthermore, it should be considered that the nutritional characteristics of the majority of eHPFs are generally inadequate for the high nutritional needs of preterm infants. Therefore, further larger trials should be carried out to confirm the efficacy of a nutritionally adequate eHPF in reducing acid GOR features and improving GOR clinical symptoms in the preterm population.

## 6. Human Milk Fortifiers and Human Milk Protein Content

In order to achieve the nutritional needs of preterm infants, human milk is usually supplemented with commercial human milk fortifiers (HMFs), which contain proteins, carbohydrates, and minerals. As stated in a systematic review [59], preterm infants fed fortified HM have improved weight gain, linear growth, and head circumference growth without experiencing major adverse effects (i.e., necrotising enterocolitis). On the other hand, HM fortification might lead to feeding intolerance, because it increases osmolality to values higher than the expected, with further increase over time [60]. Moreover, a worsening of acid GOR related to an increase in the osmolality of meals has been previously demonstrated in infants and children [61].

We have previously evaluated whether standard fortification with different amounts of HMFs (the low dose being 3% and the higher 5%) may affect GOR features in symptomatic preterm infants [62]. The standard addition of both HMF 3% and HMF 5% led to a significant increase of nonacid GORs, nonacid reflux index, and oesophageal height of reflux, while no difference was observed in acid GOR features.

So far, this is the first study to explore the effect of HMFs on GOR features in preterm infants; therefore, these preliminary data, obtained from a relatively small population, need to be validated in larger clinical trials.

## 7. Intragastric Tubes

Due to the inability of preterm infants to coordinate sucking, swallowing, and breathing, tube feeding is frequently used in NICUs. However, the presence of a tube through the gastroesophageal junction can exacerbate GOR through two different mechanisms: firstly, weakening the competence of LOS and subsequently enhancing the refluxate of gastric content into the oesophageal lumen [63] and, secondly, impairing oesophageal clearance [64]. Consistently with this assumption, Peter et al. [32] found a higher incidence of GOR, especially after the first postprandial hour, in a small cohort of preterm infants who had the MII catheter placed through the gastroesophageal junction. A similar study, evaluating the impact of two different nasogastric tubes on GOR in term neonates and older infants, found no effect with the smaller tube (8 F) but an increase of GOR in association with the larger one (12 F) [64]. Conversely, two trials [35, 65] evaluating tube versus oral feeding in symptomatic term and preterm infants found a significantly lower rate of GORs in the tube-fed group; this opposite result might be due to some methodological differences between the studies such as, for instance, the tube size or the removal of the feeding tube during the postprandial period. To avoid potential effects related to the presence of an intragastric tube in infants with GOR unable to bottle-feed, the withdrawal of the feeding tube after bolus administration might represent a feasible strategy [32]; however, before being recommended, this practice should be tested in a larger, randomized trial, in order to weigh the potential advantages against side effects (i.e., oesophageal irritation).

## 8. Pacifier Usage

Nonnutritive sucking (NNS) influences gastrointestinal functions of preterm infants, increasing the sucking reflex maturation and endorsing an earlier achievement of full oral feeding [66]; moreover, NNS is reported to slow down the intestinal transit time and to enhance weight gain [67], while no effects were observed on gastric emptying and nutrient absorption [68, 69]. A higher number of swallows are observed during NNS. It has been previously documented that swallowing elicits LOS relaxation, which is known to exacerbate the rise of gastric content into the oesophagus [70]. At the same time, however, the act of swallowing seems to promote the oesophageal clearance of refluxate [71]; therefore, a possible role of NNS on gastroesophageal reflux might be hypothesized.

The first study evaluating the role of NNS on GOR was performed by Orenstein in 1988. Term infants younger than 6 months using a pacifier underwent pH monitoring [72]; NNS was reported to influence only the frequency of GOR episodes, which was increased in the prone position and decreased in the seated position. This controversial finding, however, cannot be applied to premature babies.

So far, the efficacy of NNS by means of a pacifier on preterm infants with symptomatic GOR has been investigated only by Zhao et al. [73], who reported a faster gastric emptying as well as a reduction of GOR features (number of refluxes, reflux index, and total time at pH < 4) in infants receiving NNS; however, as GOR episodes were detected by a pH probe, the effect of NNS on nonacid GOR was not evaluated. Thus, further studies are needed to clarify the role of pacifier usage in preterm infants with pathologic GOR.

## 9. Conclusions

Gastroesophageal reflux is a common and mainly physiological condition in preterm infants. Although evidence currently available on the conservative management of GOR is still limited, a stepwise approach, with nonpharmacological strategies as the first-line treatment, is advisable in infants experiencing noncomplicated GOR. On the basis of the existing literature, body positioning can be considered the most established and safe treatment. Left-side and prone positions are effective in reducing both acid and nonacid GORs but should be limited to hospitalized preterm infants due to the higher risks of SIDS. Improvements can also be obtained by dietary changes; particularly, a prolonged feeding duration seems to be effective in infants with predominant nonacid GOR, whereas symptomatic babies with acid GOR might benefit from frequent, small feeds. Extensively hydrolysed formulas are found to improve acid GOR features, while both formula and human milk thickening resulted to be ineffective and potentially harmful. Finally, the role of nonnutritive sucking and intragastric tubes is still controversial, thereby needing to be further investigated in larger trials.
